# CNDP2: An Enzyme Linking Metabolism and Cardiovascular Diseases?

**DOI:** 10.1007/s12265-024-10560-4

**Published:** 2024-09-30

**Authors:** Moizle Grace Castro Ocariza, Louise Nancy Paton, Evelyn Mary Templeton, Christopher Joseph Pemberton, Anna Pauline Pilbrow, Sarah Appleby

**Affiliations:** Department of Medicine, Christchurch Heart Institute, University of Otago (Christchurch), Christchurch, New Zealand

**Keywords:** Carnosine dipeptidase II, CNDP2, Lac-Phe, Lactate, Lactate metabolism, Cardiovascular disease, Lactic acid

## Abstract

The heart requires a substantial amount of energy to function, utilising various substrates including lipids, glucose and lactate as energy sources. In times of increased stress, lactate becomes the primary energy source of the heart, but persistently elevated lactate levels are linked to poor patient outcomes and increased mortality. Recently, carnosine dipeptidase II (CNDP2) was discovered to catalyse the formation of Lac-Phe, an exercise-induced metabolite derived from lactate, which has been shown to suppress appetite in mice and reduce adipose tissue in humans. This review discusses CNDP2, including its role in lactate clearance, carnosine hydrolysis, oxidative stress regulation, and involvement in metabolite regulation. The association between CNDP2 and cardiometabolic and renal diseases is also explored, and knowledge gaps are highlighted. CNDP2 appears to be a complex participant in human physiological processes and disease, necessitating additional research to unveil its functions and potential therapeutic applications.

**Graphical Abstract:**

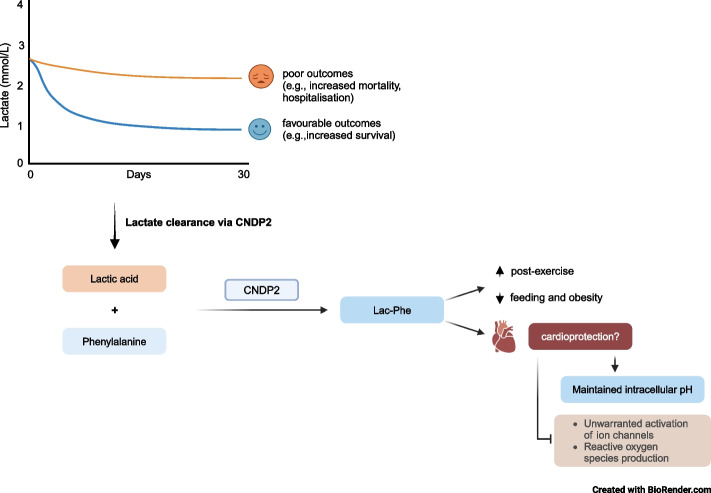

**Supplementary Information:**

The online version contains supplementary material available at 10.1007/s12265-024-10560-4.

## Introduction

The heart has a high energy demand to sustain its continuous contractile activity. It is highly adaptive and capable of metabolising various substrates as energy sources including lipids, carbohydrates, and metabolites such as lactate, pyruvate, and ketone bodies [1]. At rest or minimal work, the heart utilises lipids as the primary fuel source but switches to glucose and lactate when there is insufficient oxygen [1]. This metabolic flexibility enables the heart to sustain its output in response to stressors such as exercise, cardiovascular diseases (CVDs, e.g., myocardial infarction (MI) and heart failure) [2], and shock states [3].

Maintaining lactate homeostasis is essential for cardiac function. Lactate is produced from pyruvate during anaerobic glycolysis, particularly during high-intensity exercise or under conditions of limited oxygen supply [2, 4]. Monocarboxylate transporters (MCTs) 1 to 4 of the solute carrier family 16 facilitate the proton coupling and bidirectional transport of lactate [5]. MCT1 is involved in both the import and export of lactate depending on lactate concentration, while MCT4 is mainly involved in lactate export [2]. MCT2 is very similar to MCT1 while MCT3 is similar to MCT4 [4].

Blood lactate concentration is tightly regulated, with net lactate uptake occurring in tissues [6]. Uptake is balanced by clearance, which occurs primarily in the liver and kidneys via mitochondrial oxidation, conversion to glucose, and renal excretion [1, 6, 7]. The conversion of lactate into glucose and the presence of the mitochondrial lactate oxidation complex (mLOC) in tissues such as the heart, allows the use of lactate as an energy source [1, 6]. However, despite lactate being a primary cardiac energy source during stress, persistent concentrations above normal (≥ 2 mmol/L) have been associated with worse prognosis in critical illnesses such as CVDs and septic shock [8–15]. For example, in the context of MI, elevated lactate concentrations above 1.8 mmol/L were associated with a higher 30-day mortality rate (6.5%) compared with individuals with lower blood lactate concentrations (1.5–2%) [8]. Additionally, lactate accumulation has been linked to irreversible myocardial damage, with MI patients who have lactate > 2.5 mmol/L having a larger myocardial infarct compared to patients with lactate ≤ 2.5 mmol/L [9]. Elevated blood lactate concentrations are also associated with increased 180-day mortality and hospitalisation in patients with acute heart failure [10, 11]. Further, increased lactate clearance in MI and cardiogenic shock has been suggested to predict favourable outcomes, including better survival and neurological outcomes and improved response to percutaneous coronary intervention [8, 12–14], although the mechanisms underlying these relationships are unclear.

Recently, the enzyme carnosine dipeptidase II (CNDP2) was discovered to catalyse the condensation reaction of lactic acid (Lac) and phenylalanine (Phe) through reverse proteolysis [16]. The formation of Lac-Phe is thermodynamically unfavourable but high intracellular lactate and phenylalanine concentrations (micromolar to millimolar) permit the reaction to occur [17]. Genetic ablation of the *Cndp2* gene in mice leads to significant reductions in Lac-Phe, suggesting a direct link between CNDP2 and Lac-Phe formation [16, 17]. Given that lactate concentration can increase following a cardiovascular event, the conversion of lactate into Lac-Phe by CNDP2 may serve as a mechanism for lactate clearance. Understanding this process could provide valuable insight into the relationships between CNDP2, lactate concentrations and cardiovascular outcomes.

Accordingly, this review aims to outline the current knowledge on CNDP2 including its discovery, potential physiological functions including lactate clearance, and association with diseases.

## Discovery and Characterisation of CNDP2

CNDP2 (also known as CN2; CPGL; PEPA; HsT2298; HEL-S-13) was isolated from multiple tissues in 1985 in the bid to characterise the human tissue carnosinase [18]. Carnosinases hydrolyse carnosine, a compound with pH-buffering and anti-oxidant capabilities, and were discovered in porcine kidneys in 1949 [18, 19]. In 2003, genes encoding two human carnosinases (*CN1* and *CN2*) were discovered [20]. *CN1* (now called *CNDP1*) codes for the carnosinase isoform found in serum while *CN2* (now known as *CNDP2*) codes for the tissue carnosinase [19]. *CNDP1* and *CNDP2* are both located on chromosome 18 [19].

CNDP2 localises to the cytoplasm and nucleoplasm and is expressed ubiquitously, with the highest expression in the kidney, liver, spleen, and cerebral cortex [18, 20, 21]. Currently, it is unclear whether CNDP2 is present in the heart due to conflicting results by Lenney et al. [18] and Teufel et al. [20]. While Lenney et al. [18] reported CNDP2 activity, Teufel et al. [20] documented mRNA expression but observed no protein presence. Consistent with Teufel et al., the Genotype-Tissue Expression (GTEx) database, which contains whole genome data across multiple tissues, reports *CNDP2* gene expression in the left ventricle [22]. To date, however, no studies, including large-scale proteomics initiatives such as the Human Protein Atlas [21], have reported CNDP2 protein in the heart. A recent study, however, has identified CNDP2 in extracellular vesicles derived from myotubes treated with electrical pulse stimulation, a model of exercise, suggesting that contracting skeletal muscles may serve as a potential origin of circulating CNDP2 [23]. Thus, cardiac CNDP2 may originate from organs such as the liver or kidney and get transported into the heart via extracellular vesicles.

CNDP2 is a non-specific dipeptidase that exists as a ~ 90 kDa homodimer and belongs to the M20 metallopeptidases family [18, 20]. CNDP2 cleaves a wide variety of substrates including the dipeptides His-Ala, Gly-His, Gly-Leu, Thr-Thr and Thr-Ser/Ser-Thr and requires manganese ions for full activation, although cobalt, cadmium and zinc can also activate it to a certain extent [18, 20, 24, 25]. CNDP2 is inhibited by bestatin and other leucine-containing compounds and phenylmethane-sulphonyl fluoride [18].

## CNDP2 Orthologues

Orthologues of CNDP2 exist in several other species including horses, mice, fruit flies, yeast, and silk moth (Table 1), which have been used as models to elucidate the function of human CNDP2 [17, 24, 26–30]. So far CNDP2 orthologues are involved in Lac-Phe formation [17], oxidative response [26, 28, 29], hydrolysis of dipeptides [24, 26, 29], sensitivity to toxic agents [30], and may even be involved in nuclear processes [27].

**Table 1:** Orthologues of CNDP2 and their functions

Species	Gene	Protein	Protein sequence identity (%) to human CNDP2 [31, 32]	Experimental evidence	Reference
*Equus caballus* Horse	*CNDP2*	CNDP2	90.5	Metabolomics using mass spectrometry (MS) on pre- and post-race plasma from horses showed that the most significant exercise-inducible metabolite had a mass-to-charge ratio of 236.0928, which corresponded to Lac-Phe.	[17]
*Mus musculus* Mouse	*Cndp2*	CNDP2	91.2	Lac-Phe was found to be the metabolite most significantly induced by exercise in an untargeted metabolic analysis of pre- and post-exercise plasma using MS. Ablation of CNPD2 in mice led to elevated levels of threonyl dipeptides in multiple tissues and blood. *Cndp2* mutant mice suffered kidney damage induced by acetaminophen overdose, which depletes glutathione, a cellular antioxidant.	[17, 24, 26]
*Drosophila melanogaster* Fruit fly	*Cndp2*	CNDP2	62.8	Immunostaining showed CNDP2 localised in the cytoplasm and nucleus where it bound to the chromatin of polytene chromosomes, suggesting it might have a nuclear role (e.g., DNA replication, transcription, and repair). *Cndp2* mutant flies had shorter lifespans and increased sensitivity to paraquat or hydrogen peroxide-induced stress.	[27, 28]
*Saccharomyces cerevisiae* Yeast	*dug1*	Dug1p	51.7	Knockdown of *Dug1p* led to reduced cleavage of cysteine-glycine dipeptides. Expression of human CNDP2 restored cysteine-glycine cleavage activity to normal.	[29]
*Bombyx mori* Domestic silk moth	*Cndp2*	Cndp2	58.7	CNDP2 expression increased in larvae treated with zinc oxide, an agent that causes reduced cell viability, cell damage and cytotoxicity.	[30]

## Association between CNDP2, Lac-Phe, and Cardiovascular Homeostasis

Under stress, lactate can account for up to 60% of the heart’s energy supply [33]. This increase in lactate reliance is reflected by the increase in expression of the mLOC genes, specifically, MCT1, MCT4, and lactate dehydrogenase [1]. However, while lactate is an important energy source, persistently high lactate concentration is associated with poor prognosis, potentially through participation in processes that influence the cardiovascular system, as recently reviewed by Wu et al. [4] and Ouyang et al. [2]. For example, lactate can influence gene expression and post-translational modification of proteins (lactylation), promote inflammatory cytokine production and alter cardiac haemodynamics and electrophysiology [2, 4]. Further, the proteins involved in lactate transport and metabolism have also been implicated in CVD [2, 4, 34, 35]. For example, Dai et al. found that lactate dehydrogenase A (LDHA), an enzyme which converts pyruvate into lactate, was required for adaptive cardiomyocyte growth in response to increased ventricular wall stress [34]. They also found that lactate supplementation in LDHA-deficient cardiomyocytes rescued defective growth [34]. Meanwhile, another study found that MCTs may contribute to post-MI cardiac fibrosis by importing lactate. Fan et al. observed that increased intracellular lactate promoted Snail1 lactylation, which activated transforming growth factor beta signalling, promoting endothelial-to-mesenchymal transition (EndoMT)-mediated fibrosis [35]. Notably, they found that inhibiting MCTs or knocking down MCT1 reduced EndoMT, suggesting that MCTs contribute to EndoMT by importing lactate. [35]. Thus, it is vital that physiological lactate concentration is maintained. CNDP2 may support this by converting lactate into Lac-Phe.

Lac-Phe itself may also exert cardioprotective effects. A lack of physical activity is a major contributor to poor cardiovascular health, while regular exercise is associated with improved heart health and an overall decrease of CVD risk [36]. Mechanistic factors linking these improvements have been hard to identify, but recently, Lac-Phe was found to suppress appetite in obese mice [17]. Importantly, post-exercise, Lac-Phe concentrations in the blood were observed to increase markedly (up to 2 μM) in humans, mice, and horses [17]. Further, individuals who produce higher Lac-Phe post-exercise have a greater adipose tissue reduction compared with those who produce lower Lac-Phe despite similar relative cardiorespiratory intensities over 8 weeks [37]. These discoveries are exciting given the link between obesity and CVD risk. Adipose tissues release adipokines which have immunomodulating, metabolic, and cardiovascular effects [38]. Obesity can lead to adipose tissue dysfunction which can promote systemic inflammation and insulin resistance which impacts glucose metabolism [38].

Lac-Phe also mediates the biological weight loss-inducing effects of metformin in diabetic patients [39, 40]. A recent study observed that Lac-Phe concentration was found to acutely increase in response to metformin administration in patients with Type II diabetes [39]. Meanwhile, in a mouse model, the gut epithelium was found to be the main in vivo source of circulating CNDP2 and genetic ablation of *Cndp2* in male mice rendered them resistant to metformin’s anti-obesity effects [40]. As diabetes is a known major risk factor for CVD, these recent findings suggest that both CNDP2 and Lac-Phe could contribute to reducing CVD risk by promoting weight loss and improving glycaemic profile.

## Potential functions of CNDP2

### Cardioprotection

**Fig. 1:**
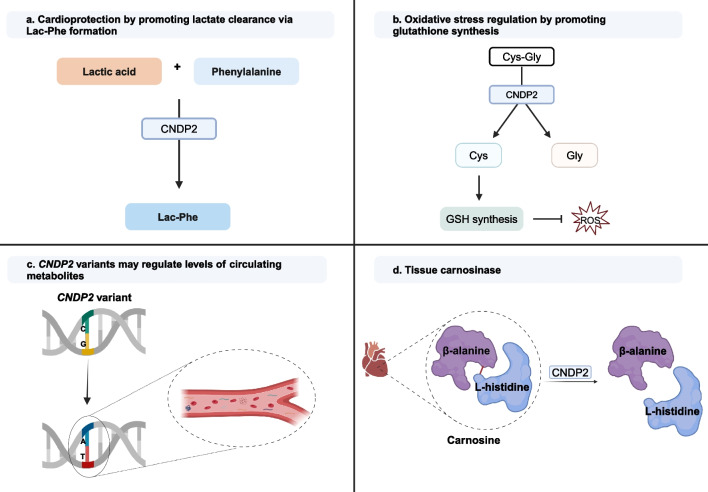
Potential functions of CNDP2.** a** CNDP2 may be cardioprotective by promoting lactate clearance.** b** CNDP2 promotes glutathione synthesis by cleaving Cys-Gly dipeptides. **c** Variations in the *CNDP2* gene are associated with circulating metabolite levels. **d** CNDP2 cleaves carnosine into its constituents. *CNDP2* carnosine dipeptidase II, *Cys* cysteine, *Gly* glycine, *GSH* glutathione, *ROS* reactive oxygen species. Created with BioRender

Historically, lactate has been regarded as a passive participant in biological processes, but the increasing number of studies reporting the association of increased lactate levels, impaired lactate clearance, and poor prognosis suggest otherwise [8–14]. The exact mechanisms as to how elevated lactate entails poor prognosis are unclear, but potential mechanisms include the up-regulation of MCT1 [41–43], decrease in intracellular pH [44–46], and activation of cardiac ATP-sensitive potassium (K_ATP_) channels [47]. In these contexts, CNDP2 could be cardioprotective by promoting lactate clearance via Lac-Phe formation (Fig. 1a).

Lactate transporters are expressed on both the sarcolemma and the mitochondrial inner membrane [48]. Increased MCT1 expression due to increased lactate has been observed in animal models of MI-induced chronic heart failure [41] and chronic myocardial volume overload [42]. The increased influx of lactate into the cell during a cardiovascular event is a compensatory mechanism aimed at providing the cell with sufficient energy [41]. However, increased mitochondrial lactate oxidation could lead to increased reactive oxygen species (ROS) production and apoptosis [43]. For example, in a study performed on right atrial appendage tissues of atrial fibrillation (AF) and sinus rhythm patients matched with normal controls, not only did the AF patients have increased MCT1, but they also had increased oxidative stress and increased expression of mitochondrial apoptotic proteins (e.g. cytochrome c, cleaved caspases 3 and 9) [43].

The accumulation of lactic acid, which dissociates into a lactate anion and H^+^ ion [49], can lead to decreased pH, which can affect cardiac contractility [44, 45] and even cause apoptosis [46]. Cardiac contraction occurs when intracellular Ca^2+^ binds with troponin [50]. However, decreased intracellular pH has been shown to reduce the binding of Ca^2+^ with troponin and decrease the probability of the opening of the sarcoplasmic reticulum, the intracellular Ca^2+^ store, leading to decreased contractility [44, 45]. Meanwhile, in rat cardiac myocytes exposed to hypoxia, lactate promoted apoptosis by stabilising BNIP3, a protein that translocates into the mitochondrial membrane and causes the release of apoptotic molecules via the mitochondrial transition permeability pore [46].

During a cardiovascular event, cardiac intracellular energy (ATP) decreases from 8 mM to 2–5 mM [47, 51]. Using guinea pig ventricular myocytes, Keung and Li found that at 2–5 mM ATP, elevated lactate concentration (20 mM) can activate K_ATP_ channels [47], which are inactive during normal physiological conditions [52]. Contraction requires a huge amount of energy, so the activation of K_ATP_ channels in times of energy crisis is regarded as an energy preservation mechanism as they cause action potential shortening which inhibits contraction [53]. However, while this activation may be compensatory when there is decreased ATP, prolonged activation of K_ATP_ channels could predispose individuals to arrhythmia. This is because when an action potential is shortened, the heart can initiate new contractions more rapidly, leading to an increased heart rate which could become erratic [54]. Given that lactate has the ability to activate K_ATP_ channels, sustained elevation of lactate could be harmful.

Taken together, through a potential to maintain lactate and H^+^ at physiological levels, CNDP2 might have cardioprotective effects by converting lactate to Lac-Phe.

### Oxidative Stress Regulation

Oxidative stress, caused by the overproduction of ROS or the depletion of cellular antioxidants, contributes to heart injury. Cardiomyocytes are particularly vulnerable due to their high mitochondrial content, which are both a source and a target of cellular oxidants [55]. Glutathione (GSH), an important antioxidant, plays a crucial role in mitigating oxidative stress and its depletion can lead to cell death, including iron-dependent ferroptosis [55]. Cysteine (Cys) is a key component of GSH and limited Cys supply can limit GSH synthesis [56]. In the GSH cycle, GSH is cleaved extracellularly into γ-glutamyl moiety and cysteine-glycine (Cys-Gly) dipeptide [56]. The Cys-Gly dipeptide can either be further cleaved by extracellular dipeptidases into Cys and Gly and imported via the neutral amino acid transporter or imported directly into the cell by the dipeptide transporter PepT2 and cleaved intracellularly [56]. Studies by Kaur et al. [29] and Kobayashi et al. [26] suggest that CNDP2 has the potential to promote recycling of Cys for GSH synthesis by cleaving Cys-Gly dipeptides (Fig. 1b). Kaur et al. demonstrated that human CNDP2 can restore Cys-Gly dipeptidase activity in yeast lacking Dug1p, the yeast counterpart of CNDP2, which also localises in the cytosol [29]. Meanwhile, Kobayashi et al. showed that CNDP2 protects cells when Cys is insufficient by hydrolysing imported Cys-Gly dipeptides [26].

The potential role of CNDP2 as a regulator of oxidative stress was also shown in mice [26] and fruit flies [28]. In mice, induction of acetaminophen overdose, which causes damage by depleting GSH stores, caused kidney damage in CNDP2 mutant mice [26]. Measurement of blood urea nitrogen, a marker for kidney damage, was elevated in the CNDP2 mutant mice but not in the control mice [26]. Similarly, fruit flies that lacked CNDP2 were observed to have a shorter lifespan due to increased sensitivity to paraquat or hydrogen peroxide-induced oxidative stress [28].

### Metabolite Regulation

CNDP2 may be involved in metabolite regulation (Fig. 1c). Several genetic variants [single nucleotide polymorphisms (SNPs)] in the *CNDP2* gene are reported in the genome wide association studies (GWAS) catalog as being strongly associated with the metabolites valyglycine, leucyalanine, valylleucine, and gamma-glutamyl-2-aminobutyrate (see supplementary Table 1) [57]. CNDP2 was also found to cleave threonyl dipeptides (threonyl-threonyl and threonyl-serine/serine-threonyl isomers) in a mouse model [24].

### Tissue Carnosinase

The function of CNDP2 as a tissue carnosinase (β-Ala-His dipeptidase) remains a topic of ongoing debate (Fig. 1. d). The GWAS catalog reports two SNPs in the *CNDP2* gene (rs11664131 and rs8084058) associated with an increase in β-Ala-His dipeptidase levels [57]. However, experiments performed in vitro have shown that CNDP2 has very minimal activity against carnosine at physiological pH, suggesting that carnosine may not be its physiological substrate, although there is consensus that as pH increases, CNDP2 activity increases [18, 20, 58]. Additionally, in a mouse model, overexpression of CNDP2 in the liver did not alter tissue carnosine levels, further showing that CNDP2 may not be a carnosinase [24]. Carnosine levels in the heart are also relatively low (0.1 mmol/kg wet weight) [59], limiting its contribution to pH-buffering and oxidative stress response. Additionally, cardiomyocytes have an intracellular pH of ~ 7.2 [59] while the optimal pH for the carnosinase activity of CNDP2 is 9.5 [18, 20]. As such, the carnosinase activity of CNDP2 in cardiomyocytes would be expected to be minimal.

## CNDP2 in Cardiometabolic and Renal Diseases

Although the physiological role of CNDP2 in humans is incompletely understood it may play a role in the development of cardiometabolic and renal diseases. Comparison of gene expression between normotensive and hypertensive mice models showed that *Cndp2*, alongside the histidine decarboxylase gene (*Hdc*) was upregulated in the kidneys of the hypertensive mice [60]. The authors suggested that Hdc and Cndp2 are involved in sympathetic activity in that Cndp2 degrades carnosine into histidine, which Hdc converts to histamine, a neurotransmitter involved in regulating renal sympathetic activity and response to inflammation [60]. However, given that the carnosinase activity of CNDP2 has not been elucidated, a potential alternative mechanism through which CNDP2 contributes to regulating hypertension is via Lac-Phe formation, since increased lactate concentrations have been associated with a higher risk of hypertension [4].

In diabetic retinopathy, CNDP2 may contribute through Lac-Phe [61]. In a recent study aimed at identifying metabolites associated with diabetic retinopathy, *N*-lactoyl-amino acids including Lac-Phe were found to increase the risk of diabetic retinopathy [61]. CNDP2 may also mediate the development of post-transplantation diabetes mellitus (PTDM), a common complication of kidney transplant [62]. In their study, Kipp et al. found that CNDP2 was overexpressed in the glomeruli of PTDM patients compared to those with pre-existing Type II diabetes mellitus [62].

Using a mouse model, *Cndp2* was found to be downregulated in lupus nephritis, an inflammation of the kidneys [63]. Along with other differentially expressed genes (*Kynu*, *Spidr*, *Gbp3*, *Cbr1*, and *Cyp4b1*), *Cndp2* was hypothesised to regulate inflammation although their regulatory mechanism is unknown [63]. As lactate is elevated in inflamed tissues and can exacerbate the inflammatory response [64], CNDP2 may be regulating inflammation through Lac-Phe formation.

Several common SNPs in the *CNDP2* gene (rs373836366, rs780772968, rs4891558, and rs7577) have been linked to susceptibility to obesity and renal disease [17, 65, 66]. Genome-wide association studies have identified two missense SNPs in *CNDP2* (rs373836366 and rs780772968) associated with body mass index [17] and an intron variant (rs4891558) associated with obesity in individuals with high carbohydrate intake and low carotene intake in a Japanese population [65]. In a large candidate gene study, the SNP rs7577 was associated with altered kidney function and an increased risk of developing nephropathy in 4,888 Swedish patients with Type II diabetes, particularly in women [66]. While functional experiments are yet to confirm these associations, rs7577 is hypothesised to increase the carnosinase activity of CNDP2 [66]. However, as mentioned previously, there is conflicting evidence on whether CNDP2 can cleave carnosine at physiological pH [18, 20, 58].

## CNDP2 in other Diseases

Altered protein expression of CNDP2 has been observed in various cancers [67–76] and Parkinson’s disease [77]. The exact mechanisms of how CNDP2 drives (or inhibits) cancer and Parkinson’s disease development have yet to be established. In cancer, CNDP2 has been observed to activate the PI3K/AKT [67] and MAPK signalling pathways [72], upregulate key cell cycle proteins (e.g. cyclin E, cyclin B1) [68] and interact with pepsinogen C [73]. Meanwhile, CNDP2 is hypothesised to be involved in Parkinson’s disease through oxidative stress, protein aggregation, or inflammation [77].

## Future Directions

To gain a comprehensive understanding of the role of CNDP2 in the human heart and cardiovascular disease pathophysiology, additional research is needed. Establishing the presence of CNDP2 in the heart is a key first step. Additionally, the regulatory mechanisms, including the signals that trigger its activation and repression, are currently unknown and need to be elucidated. Potential regulatory microRNAs [23, 78] have been identified, but their roles and clinical relevance require validation in large cohorts. In addition to identifying its regulators, the proteins that interact with CNDP2 also need to be identified. This will clarify the role of CNDP2 in critical signalling pathways such as the PI3K pathway.

The role of CNDP2 in lactate clearance needs to be confirmed. If CNDP2 does play a role in this process, subsequent research should focus on developing strategies to increase its activity, potentially leading to more efficient lactate clearance. Further, the involvement of CNDP2 in CVD pathophysiology remains to be elucidated. Future research should focus on how changes in expression or activity contribute to the development and progression of CVD. This includes exploring potential associations between CNDP2 and specific cardiovascular conditions, such as heart failure and myocardial infarction.

Lastly, investigating CNDP2 as a potential therapeutic target or biomarker for CVD could have significant clinical implications. Future research should assess whether CNDP2 concentrations correlate with disease severity or prognosis, potentially leading to new opportunities for diagnostic, prognostic and therapeutic strategies.

## Conclusion

CNDP2 may play a key role in CVD through its potential role in lactate clearance. Elevated lactate levels are linked to poor prognosis in CVD, and CNDP2 may help maintain homeostatic balance by reducing these levels. The aberrant expression of CNDP2 and its associations with disease traits and circulating metabolites highlight its physiological relevance. Further investigation is essential to understand the role of CNDP2 in human physiology and disease pathophysiology.

## Supplementary Information

Below is the link to the electronic supplementary material.

Supplementary file1 (PDF 86 KB)

## Abbreviations

CNDP2: Carnosine dipeptidase II
CNDP1: Carnosine dipeptidase I
CVD: Cardiovascular diseases
MI: Myocardial infarction
MCT: Monocarboxylate transporter
K_ATP_ channel: Cardiac ATP-sensitive potassium channel
Cys: Cysteine
Gly: Glycine
ROS: Reactive oxygen species
GSH: Glutathione
SNP: Single nucleotide polymorphisms
GWAS: Genome wide association studies
GTEx: Genotype-Tissue Expression
mLOC: Mitochondrial lactate oxidation complex
PTDM: Post-transplantation diabetes mellitus
LDHA: Lactate dehydrogenase A
