# Supplementary material for: CNDP2: An Enzyme Linking Metabolism and Cardiovascular Diseases?
Source: J Cardiovasc Transl Res. 2024 Sep 30;18(1):48–57. doi: 10.1007/s12265-024-10560-4 (PMC11885389; doi:10.1007/s12265-024-10560-4)
Supplement: Supplementary file 1 — Supplementary file1 (PDF 86 KB) [file 12265_2024_10560_MOESM1_ESM.pdf]

Supplementary Table 1. *CNDP2* genetic variants associated with circulating metabolite concentrations.

Metabolite	Associated variant	Effect on metabolite measurement	Variant type
Valylglycine	rs12964619	Increase	intron
	rs2241509		non-coding transcript exon
	rs2278158		non-coding transcript exon
	rs2278159		stop gain
	rs2278161		missense
	rs2303463		missense
	rs3764509		5-prime UTR
	rs6566811		missense
	rs734559		intron
	rs12964619		intron
	rs2241509		non-coding transcript exon
	rs2278158		non-coding transcript exon
	rs2278159		stop gain
	rs2278161		missense

	rs2303463		missense
	rs3764509		5-prime UTR
	rs6566811		missense
	rs734559		intron
Leucylglycine	rs734559	Increase	intron
	rs2241509		non-coding transcript exon
	rs2278159		stop gain
	rs2278161		missense
	rs2303463		missense
	rs3764509		5-prime UTR
	rs2241509		non-coding transcript exon
	rs2278159		stop gain
	rs2278161		missense
	rs2303463		missense
	rs3764509		5-prime UTR
	rs734559		intron

Gamma-glutamyl-2-aminobutyrate	rs12964619	Increase	intron
	rs2278159		stop gain
	rs2278161		missense
	rs2303463		missense
	rs3764509		5-prime UTR
	rs734559		intron
	rs12964619		intron
	rs2278159		stop gain
	rs2278161		missense
	rs2303463		missense
	rs3764509		5-prime UTR
	rs734559		intron
Leucylalanine	rs12971120	Decrease	intron
Valylleucine	rs3829640	Decrease	non-coding transcript exon
